# Chinese Medicine Huzhen Tongfeng Formula Effectively Attenuates Gouty Arthritis by Inhibiting Arachidonic Acid Metabolism and Inflammatory Mediators

**DOI:** 10.1155/2020/6950206

**Published:** 2020-10-09

**Authors:** Jianping Deng, Zicong Wu, Chen Chen, Zhenling Zhao, Yifei Li, Zhengquan Su, Yiguang Lin, Yifei Wang, Zhiping Wang

**Affiliations:** ^1^Guangdong Provincial Engineering Center of Topical Precise Drug Delivery System, Department of Pharmaceutics, Guangdong Engineering Research Center of Natural Products and New Drugs, Guangdong Provincial University Engineering Technology Research Center of Natural Products and Drugs, Guangdong Pharmaceutical University, Guangzhou, China; ^2^Guangzhou Jinan Biomedical Research and Development Center, Guangzhou, China; ^3^College of Life Science and Technology, Jinan University, Guangzhou, China; ^4^School of Life Sciences, University of Technology Sydney, Broadway, NSW, Australia

## Abstract

The Chinese herbal medicine, Huzhen Tongfeng Formula (HZTF), derived from traditional Chinese medicine (TCM) practice, has recognized therapeutic benefits for gouty arthritis (GA). HZTF is currently in the late stage of approval process as a new anti-GA drug application. However, the underlying mechanism of HZTF as an antigout medication is unclear. In this study, we combined network pharmacology and experimental validation approaches to elucidate the mechanism of action of HZTF. First, the relative drug-disease target networks were constructed and analyzed for pathway enrichment. Potential pathways were then validated by *in vitro* and *in vivo* experiments. We found that 34 compounds from HZTF matched 181 potential drug targets. Topology analysis revealed 77 core targets of HZTF, which were highly related to gout, following screening of KEGG pathway enrichment. Further analysis demonstrated that the arachidonic acid metabolic pathway was the most relevant pathway involved in the mechanism of HZTF. Validation experiments showed that HZTF significantly inhibited the inflammatory cell infiltration into gouty joints, improved the swelling of affected joints, and increased the pain threshold. HZTF significantly reduced the transcription and production of various cytokines and inflammatory mediators *in vitro*. In particular, cyclooxygenase (COX)-1, COX-2, and 5-lipoxygenase were simultaneously downregulated. In conclusion, our study suggests that the antigout mechanism of HZTF is associated with the inhibition of the arachidonic acid pathway, resulting in the suppression of inflammatory cytokines and mediators. These findings extend our understanding of the pharmacological action of HZTF, rationalizing the application HZTF as an effective herbal therapy for GA.

## 1. Introduction

Gouty arthritis (GA) is a common type of inflammatory disease resulting from a disturbance in purine metabolism, which leads to the deposition of monosodium urate (MSU) in joints or/and tissues. GA is characterized by red, tender, hot, and tumid joints [1, 2]. Changes in life-style and dietary structure in the last few decades have led to increasing prevalence of gout worldwide [3–5]. Currently, there is no cure for gout, and clinical treatments mainly aim at relieving symptoms and preventing recurrence. Colchicine, glucocorticoids, and nonsteroidal anti-inflammatory drugs (NSAIDs) are mainly used to relieve acute GA, while allopurinol, benzbromarone, and other uric acid-lowering drugs are recommended for chronic gout [6]. Although these medications are relatively effective in controlling symptoms, side effects and relapse after withdrawal are problematic, leading to negative impacts on clinical outcomes [6–9]. Therefore, there is an unmet need to develop new antigout drugs that are more efficacious with less side effects [10].

Traditional Chinese herbal medicine (TCM) has been used for the prevention and treatment of gout for many centuries in China [11, 12]. There is increasing evidence to show that a number of TCMs have multiple effects, including anti-inflammatory, analgesic, lowering hyperuricemic, and renal protective effects, with the potential for wider use in the management of gouty conditions [11–16]. It has been reported that TCM Huzhang Tongfeng Granule promotes the excretion of urine uric acid and improves inflammatory symptoms and signs of gouty arthritis patients in the acute phase [17]. The TCM preparation of Rebixiao granules was demonstrated to be more effective in controlling recurrence, signs, and the symptoms of patients with acute gouty arthritis compared with diclofenac sodium [18]. Leucas zeylanica, a herb commonly use in anti-inflammatory and antigout remedy, was found to possess potent inhibitory effect on 5-LO, mPGES-1, and XO [15]. More convincingly, randomized clinical trials showed that TCM formulas Weicao capsule, Chuanhu formula, and modified Simiao decoction were more effective than the conventional antigout treatment, with improvement in the adverse side effect profiles [19–21].

Huzhen Tongfeng Formula (HZTF) is a TCM formula for GA, developed by our team at Jinan University based on principles derived from traditional Chinese medicine (TCM) practice [22]. It is composed of 4 Chinese herbs: Polygoni Cuspidati Rhizoma et Radix (PCRR, the root and rhizome of *Polygonum cuspidatum* Sieb. et Zucc.), Ligustri Lucidi Fructus (LLF, the fruit of *Ligustrum lucidum* Ait.), Herba Plantaginis (HP, the dried whole grass of *Plantago asiatica* L.), and Nidus Vespae (NV, the honeycomb of *Polistes olivaceus* (De Geer), Polistes Japonicus Saussure, or Parapolybiavaria Fabricius). Clinical data has shown that HZTF significantly suppresses the inflammation and pain in gouty arthritis patients, with a response rate of 96% [22]. No hypouricemic effect was found associated with the use of HZTF in these studies. In a separate clinical study involving 287 GA patients, pain reduction was observed in 86.11% of patients, and adverse events were recorded in only 2.78% of patients. Since its prominent antigout effect with minimal side effects and its suitability for long term use, HZTF has been considered as a new antiarthritis drug by the National Medical Products Administration (NMPA) of China under the category of new TCM natural drug. Required clinical trials (registered number: CTR20150783and CTR20131159) have been completed. Although HZTF is now at its late stage of NMPA approval process based on its improved efficacy in GA patients, its mechanism of action as an anti-GA drug is unknown.

Since HZTF is composed of multiple compounds, compiled from 4 TCM herbs, the usual approach employing the paradigm of “one molecule, one drug” may not be a suitable pathway to investigate the mechanism of action of HZTF. Alternatively, a network pharmacological approach has the potential to uncover the underlying complex relationship between an herbal formula and the breadth of GA targets. Previous studies have demonstrated network pharmacology is capable of handling the holistic concept and syndrome differentiation to discover the synergy between the active ingredients [23–26]. In addition, a systematic and multilayered “drug-gene-disease” network, designed to discover the synergy between the active ingredients and components of the TCM, may further predict the pharmacological mechanism. This approach would be helpful in the study of active substances, compatible regularity, and multichannel systemic regulation mechanisms of Chinese medicinal compounds to promote the development of new drugs for multimolecular and multitarget therapy [24, 25].

**Figure 1:**
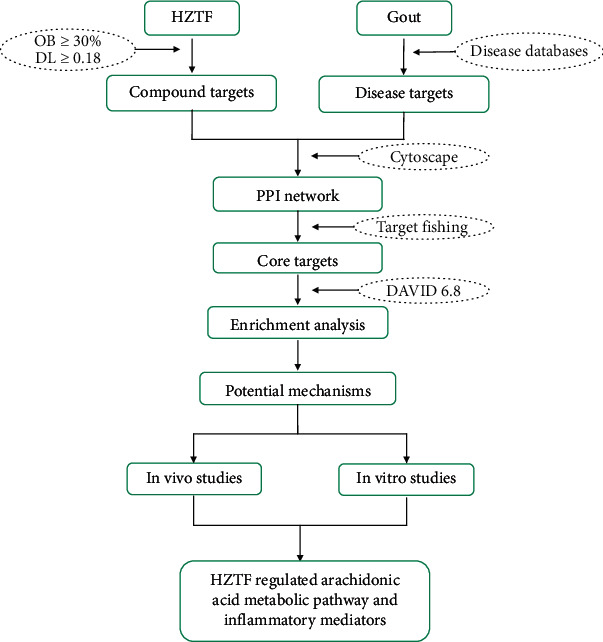
Flowchart of study design—based on an integration strategy of network pharmacology and experimental validation.

In this study, we combined network pharmacology and experimental validation approaches, as outlined in Figure 1, to illustrate the mechanism of action of HZTF, to uncover the compound–compound targets and compound-disease target networks, and the potential signaling pathways involved in the pharmacological action of HZTF as antigout therapy.

## 2. Materials and Methods

### 2.1. HZTF Ingredients and Related Targets Screening

Compounds of PCRR, LLF, and HP were obtained according to selection criteria (drug − likeness (DL) ≥ 0.18 and oral bioavailability (OB) ≥ 30) from the TCM Systems Pharmacology Database and Analysis Platform (TCMSP)^1^ and related targets [27]. In addition, the ingredients of NV obtained from the Reference Handbook for Chinese Pharmacopeia (Volume I)-Modern Analysis Technology for Evaluating the Quality of Traditional Chinese Medicine. The potential targets were obtained from Swiss Target Prediction2. By using the platform to query the corresponding target of the compound, Uniprot normalizes the gene information and eliminates the gene without the human sample Uniprot ID.

### 2.2. Collection of Potential Targets for Gout

Gout-associated targets were obtained from the following databases using “Gout” as the keyword: [1] Therapeutic Targets Database^3^, [2] DrugBank^4^, [3] Comparative Toxicogenomics Database^5^, [4] The Online Mendelian Inheritance in Man (OMIM) database^6^, and PubMed^7^. The platform was used to search for “gout”-related target genes, Uniprot standardized gene information, and the gene without the human sample, Uniprot ID, was excluded.

### 2.3. Systems Network Construction and Analysis

Protein-protein interactions greatly influence the biological process of the organism, including signal transduction, immunoregulation, and cell proliferation [28]. To better elucidate the relationship between HZTF and gout, a compound-target-disease network was built with the protein-protein interaction (PPI) information, which was visualized and analyzed by Cytoscape v.2.5.1 (National Institute of General Medical Sciences, United States). The network topology parameters, including Degree, Betweenness Centrality, Average Shortest Path Length, and Closeness Centrality, were determined [29]. The degree of a node in the network indicates the number of nodes directly interacting with the node. The greater the degree, the more biological functions that node participates in, and the greater its importance in the network.

### 2.4. Gene Ontology and Pathway Analysis

To analyze and obtain the main function and the enriched-pathway of the target genes, Gene Ontology (GO) and pathway analysis were performed through the Database for Annotation, Visualization and Integrated Discovery (DAVID 6.8) [23, 30]. Briefly, a list of target gene names was entered into the database. The species was then defined as “Homo Sapiens”; the target gene name was corrected to the official name (“official gene symbol”); the threshold was set to *P* < 0.05 for GO biological process and KEGG pathway enrichment analysis. Correlation analysis results were obtained with *P* < 0.05 as the screening condition.

### 2.5. Animals and Drug

Male Japanese big ear white rabbits (weight 3.0 ± 0.5 kg) were purchased for the GA model (described in 2.6) from the Department of Laboratory Animal Science, Tongji Medical College, Huazhong University of Science and Technology, China (Certification No: YDZ 19-025). Male Kunming mice (weight 25 ± 2 g) were purchased from the Henan Experimental Animal Center, China (Certification No: 410115). Rabbits and mice were housed in a SPF-grade environment with water and food, at a constant temperature (23 ± 2°C), and humidity of 60 ± 5% in the Experimental Center of Jinan University.

HZTF was provided by the Guangzhou Jinan Biomedicine Research and Development Center Co., Ltd., (Lot: 030901). Monosodium urate crystal, xylene, and antimony potassium tartrate were purchased from Shanghai Yuanye Bio-Technology Co., Ltd, China. Dulbecco's modified Eagle's medium (DMEM) (Gibco BRL, Grand Island, NY, USA), fetal bovine serum (FBS) (Guangzhou Juyan Co., Ltd., China), 3-(4,5-dimethylthiazol-2-yl)-2,5-diphe-nyltetrazolium bromide (MTT) (Guangzhou Juyan Co.,Ltd.,China), and uric acid (SERVA, Germany) were used in this experiment. All other solvents and chemical reagents used in this study were of analytical grade and were purchased locally.

### 2.6. MSU Crystal-Induced Inflammation in Rabbits

All procedures were performed based on methods described previously [31], with slight modifications. Rabbits were randomly divided into 5 groups (control, model, and HZTF at high, median, and low doses). HZTF groups were orally administered different dosages once daily for 5 days. Thirty minutes after the 5th administration, animals were injected 0.3 mL MSU crystal suspension (100 mg/mL) at the medial side of the right ankle joint of the hind limb for the model group and HZTF groups, respectively. Rabbits were sacrificed 5 h after injection. Joint effusion was collected from anatomical knee joints, and white blood cell counts were measured.

### 2.7. Ear Edema Induced by Xylene in Mice

All procedures were executed based on methods described by Chen and colleagues [32], with modifications. The HZTF groups were orally administered different dosages once daily for 5 days. Thirty minutes after the fifth administration, 50 *μ*L of xylene was smeared on the surfaces of the right ear. After 1 h, mice were sacrificed, and both ears of each animal were collected. Using a 6 mm diameter puncher, round pieces of ear tissues were punched and weighed. The difference in weight between the left and right ears was considered to represent the degree of swelling. The study was approved by the institutional research ethics committee of the Laboratory Animal Center of Henan Province.

### 2.8. Hot Plate Test in Mice

To assess the analgesic effect of the drug, mice were orally administered different dosages of HZTF once daily for 5 days. The nociceptive threshold was evaluated as the reaction time to the hot plate (55 ± 0.5°C). The response was measured as the time to licking of the forepaws or hind paws by mice. The maximal cutoff time was set to 60 s to reduce injury to the mouse. At 30, 60, and 90 min after the fifth administration, the antinociceptive effect of HZTF was determined [33]. The study was approved by the institutional research ethics committee of the Laboratory Animal Center of Henan Province.

### 2.9. Antimony Potassium Tartrate-Induced Abdominal Writhing Test in Mice

For the antimony potassium tartrate-induced writhing experiment, mice were orally administered different dosages of HZTF once daily for 5 days. Thirty minutes after the final administration, mice were injected i.p. with 0.05% antimony potassium tartrate (0.1 mL/10 g). The number of abdominal constrictions was recorded for 20 min after the injection [34]. The study was approved by the Ethics Committee of Zhengzhou University.

### 2.10. Cell Culture

The murine macrophage cell line, RAW264.7, was obtained from the American Type Culture Collection (Manassas, VA, USA) and was propagated in DMEM high glucose medium supplemented with 10% heat-inactivated calf serum. Cells were cultured at 37°C in a 5% CO_2_ incubator for various *in vitro* studies as detailed below.

### 2.11. MTT Assay

The cytotoxic effect of HZTF on RAW264.7 cells was tested using the MTT assay following the Mosmann method [35]. Briefly, RAW264.7 cells were first plated in 96-well plates overnight; the medium was then aspirated, and subsequently, cells were treated with different concentrations of HZTF for 24 h. After the medium was removed, cells were washed and incubated with MTT for 4 h. The medium was then removed, and 100 *μ*L of DMSO was added. Absorbance was measured at 570 nm [36].

### 2.12. LPS-Induced Cell Inflammation Model

RAW264.7 mouse macrophage cells were cultured in DMEM containing 10% FBS at 37°C with 5% CO_2_ in the air. To evaluate the anti-inflammatory activities of HZTF, RAW264.7 mouse macrophages were cultured in 6-well plates overnight to reach 80% confluence. Cells were pretreated with different concentrations of HZTF (12.5, 25, and 50 *μ*g/mL) or the positive control, dexamethasone (10 *μ*g/mL). Following addition of lipopolysaccharide (LPS 100 ng/mL) and incubation, culture media and cells were collected for further experimentation.

### 2.13. RNA Isolation and Real-Time PCR

After 17 h of incubation with LPS and HZTF, cells were washed with 1× PBS. TRIzol reagent was then added for total RNA isolation. The IScript Advanced cDNA synthesis kit was used to reverse transcribe cDNA. Real-time PCR was performed on a Bio-Rad Real-Time PCR System using SYBR Green PCR Master Mix. Mouse specific primers, detailed in Table 1, were synthesized by Sangon Biotech (Shanghai, China). The following amplification parameters were used for PCR: 50°C for 2 min, 95°C for 10 min, and 40 cycles of amplification at 95°C for 15 s and 60°C for 1 min. All experiments were performed in triplicate.

### 2.14. Measurement of Inflammatory Cytokines and Mediators

RAW264.7 cells were plated at a density of 5 × 10^5^/mL on 6-well plates and pretreated with different concentrations of HZTF 1 h before treatment with LPS (100 ng/mL). After one day of incubation, the culture supernatant was collected to quantify the cytokine and NO level. Cytokine concentration was quantified using the ELISA kits from NEOBIOSCIENCE; optical density at 450 nm was determined using a microplate reader (ELx800, BioTek, USA). NO was quantified by the Griess reaction (Nitrate/Nitrite Assay Kit, Beyotime, Shanghai, China), and optical density was determined using a microplate reader set at 540 nm. All experiments were performed in triplicate.

### 2.15. Imaging and Quantification of Intracellular Reactive Oxygen Species (ROS) Generation

Briefly, dichlorodihydrofluorescein diacetate (DCFH-DA) was hydrolyzed in the cell by esterase to form DCFH, which is oxidized by ROS to generate fluorescent DCF. The RAW264.7 cell line used in this experiment was seeded onto 6-well plates at a density of 1 × 10^6^/well. Overnight, the cells were pretreated with different concentrations of HZTF for 1 h and exposed to LPS (final concentration, 100 ng/mL) and incubated in a CO_2_ incubator for 24 h. The plates were washed 3 times with free-FBS DMEM. DCFH-DA (diluted in DMEM, final concentration, 10 *μ*M) was then added and incubated for 1 h in the dark. Free dye was eluted with DMEM, and the fluorescence was measured using a fluorimeter at 488 nm excitation and emission at 520 nm (Ti-E, Nikon, Japan) [37]. All experiments were performed in triplicate.

### 2.16. Western Blot Analysis

Proteins in the RAW264.7 macrophages were extracted and adjusted to achieve the same volume and equal concentration. The protein extracts were subjected to 10% sodium dodecyl sulfate-polyacrylamide gel electrophoresis and transferred to polyvinylidene fluoride (PVDF) membranes. PVDF membranes were blocked with 5% bovine serum albumin in tris-buffered saline-Tween 20 (TBST) buffer (137 mM NaCl, 20 mM Tris, pH 7.6, with 0.1% Tween 20) for 60 min at room temperature. The PVDF membrane was washed 3 times with TBST for 10 min per wash. The membranes were then incubated overnight at 4°C with antiphospholipase A2 (cPLA2) antibody (Santa Cruz, CA, USA) at 1 : 500, anti-COX-2 antibody (Santa Cruz) at 1 : 500, anti-COX-1 antibody (Genetex, TX, USA) at 1 : 1000, antiarachidonate 5-lipoxygenase activating protein (FLAP) antibody (Genetex) at 1 : 1000, and antiarachidonate 5-lipoxygenase (5-LO) antibody (CST, MA, USA) at 1 : 1000. After 3 washes with TBST, the membranes were incubated with secondary antibody (1 : 7000) in TBST with 5% bovine serum albumin for 1 h before 3 [16] additional washes with TBST. Western blots were developed on films using the enhanced chemiluminescence technique. Quantification of bands was determined by densitometry analysis using TanonImagine. Data were normalized using *β*-actin (1 : 2000) (CST) as an internal control [29]. All experiments were performed in biological triplicate.

### 2.17. Statistical Analysis

All data are expressed as mean ± SD. Statistical analysis was performed using GraphPad Prism 7 (San Diego, CA, USA). Data among the groups were analyzed with ANOVA. A *P* value < 0.05 was considered statistically significant.

## 3. Results

### 3.1. Identification of Active Compounds in HZTF and HZTF Targets

**Figure 2:**
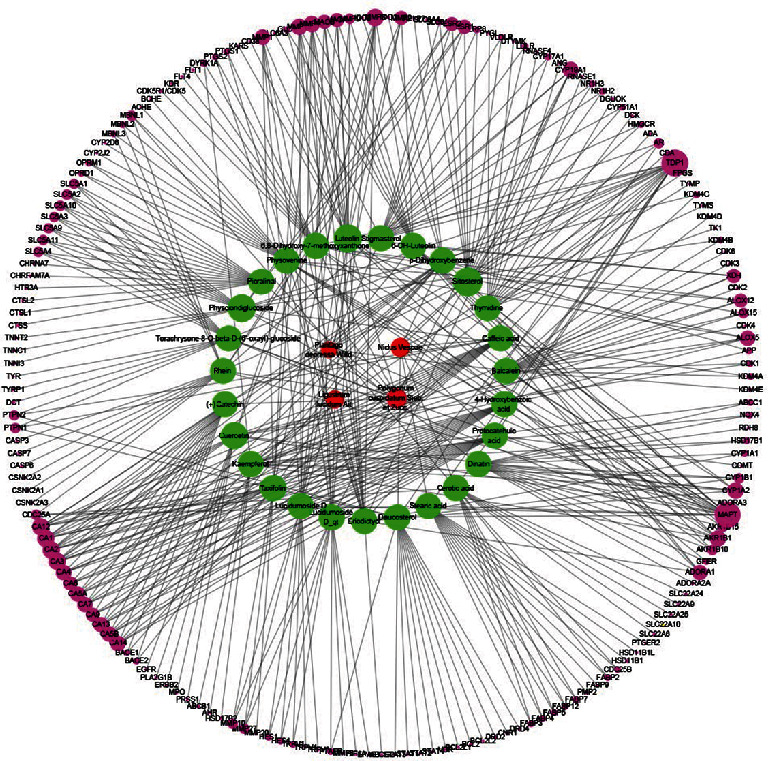
The compound–compound target network. Red, green, and purple circles represented the herbs composing HZTF, active compounds from the herbs, and targets of the compounds, respectively. The size of purple circles represented node degree value.

Using a network pharmacology approach, compounds/ingredients from each herbal medicine of the HZTF used for target identification were searched and selected based on published studies and relevant databases by the specific ADME method. As shown in Table 2, 34 active compounds from herbs of HZTF were identified and included in the study, of which, 10 compounds from PCRR, 7 from HP, 8 from LLF, and 9 from NV. Furthermore, 181 drug targets associated with the 34 active ingredients from HZTF were obtained (details in Figure 2 and Supplementary Table S1). The graph of the compound–target interactions was constructed, consisting of 212 nodes and 440 edges.

### 3.2. Construction and Analysis of Compound Target–Disease Target Network

**Figure 3:**
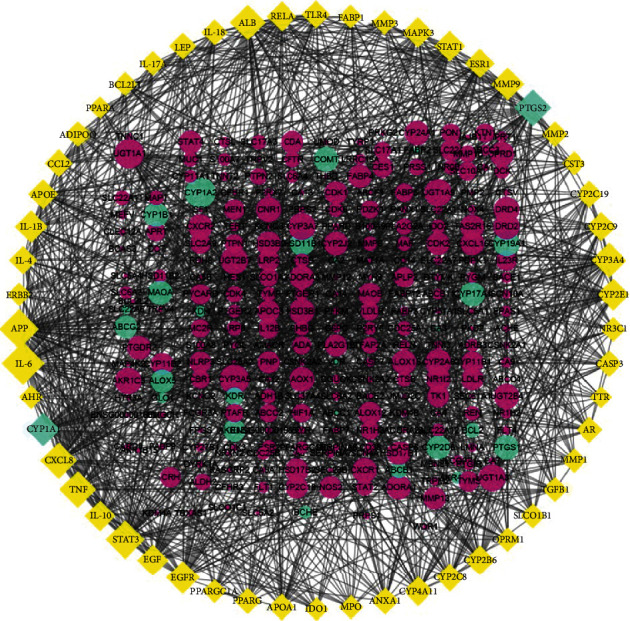
The protein-protein interaction network. Yellow diamonds and red circles represented the targets of compounds and disease, respectively. Green diamonds and green circles indicated 28 common targets. The size of circles and diamonds indicated node degree value.

Based on available data from various gouty disease databases, 224 gout-related targets were identified (Supplementary Table S2). By mapping the compound targets with these gout-related targets, 28 common targets were identified. These 28 targets and their related targets were used to build a PPI network comprising 317 nodes and 1515 edges (Figure 3). Average values for “Degree,” “Closeness,” and “Betweenness” for nodes were 6, 0.3092, and 0.0015, respectively. By screening according to specific conditions (^“^Degree^”^ ≥ 12, ^“^Closeness^”^ ≥ 0.309, ^“^Betweenness^”^ ≥ 0.002), 77 important targets were obtained, which were used for further analysis (Supplementary Table S3).

### 3.3. Analysis of Function and Pathway Enrichment

**Figure 4:**
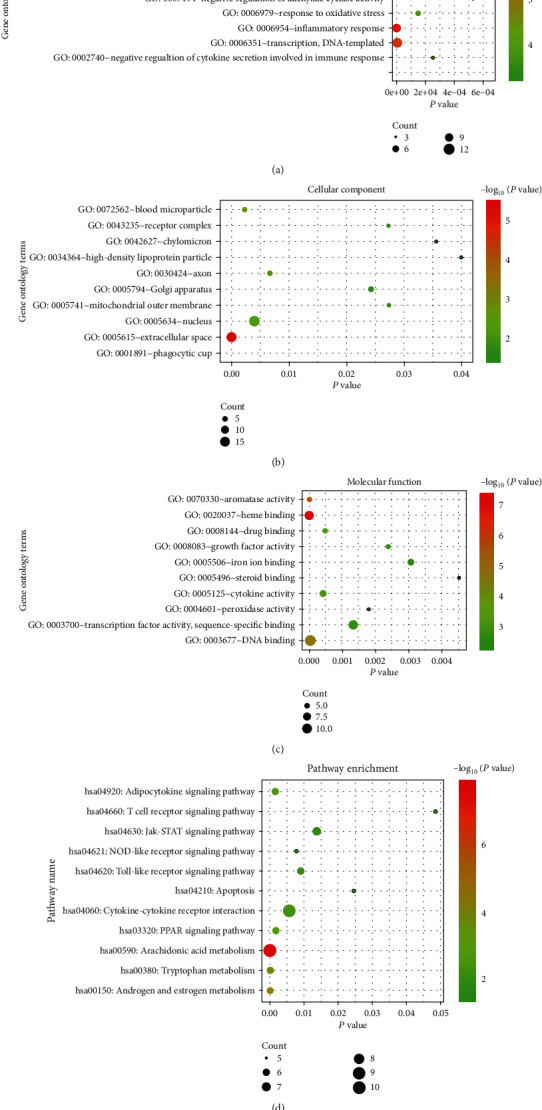
DAVID database enriched pathways and GO entries. (a) GO enrichment entries of the biological process showing the top 10. (b) GO enrichment entries of cellular components (the top 10). (c) GO enrichment entries of molecular function (the top 10). (d) KEGG pathway enrichment entries (*P* < 0.05).

For GO enrichment analysis, 77 important targets described above were entered into the DAVID database for further analysis. As a result, 153 entries (Supplementary Table S4) were found to be enriched, including various categories such as positive regulation of transcription from RNA polymerase II promoter, inflammatory response, extracellular space, DNA binding, and cytokine activity. An overview of the biological process, cellular components, and molecular function categories was illustrated with the top 10 enriched terms using a bubble (Figures 4(a)–4(c)). Further KEGG pathway enrichment analysis demonstrated that 11 pathways were identified as significant and meaningful (*P* < 0.05) pathways, including arachidonic acid metabolism, cytokine-cytokine receptor interaction, NOD-like receptor signaling pathway, and JAK-STAT signaling pathway, as shown in Figure 4(d) and Supplementary Table S4.

### 3.4. HZTF Ameliorated Acute Gout Related Inflammation In Vivo

Typical clinical presentations of acute gout mainly include articular and periarticular swelling and pain and rapid recruitment of innate immune cells into the synovium. To assess the therapeutic effect of HZTF on acute gout, the anti-inflammatory, detumescence, and analgesic effects of HZTF were examined. Following treatment with HZTF for 5 days, animals' leucocyte count in the joint effusion, ear swelling, and pain response were evaluated. We found that the number of white blood cells (WBC) in the synovium of the gout models and the HZTF groups was significantly increased compared with the control. HZTF treatments significantly lower the WBC in the synovium (Table 3). As shown in Table 4, HZTF inhibited ear swelling in a dose-dependent manner, ranging from 17.7% to 56.9%, compared to that for the untreated group. In the hot plate test in mice, HZTF significantly extended the time to withdraw from the hot plate compared to the control group. Of noted, the maximum protection of HZTF was displayed at 60 min after drug administration at a dose of 1.00 g/kg (Table 5). We also adopted the antimony potassium tartrate-induced abdominal writhing test to verify the antinociceptive effect of HZTF. The results indicated that HZTF dose-dependently decreased the number of writhing episodes in mice in comparison to the model group (Table 6).

### 3.5. Effects of HZTF on ROS Production

**Figure 5:**
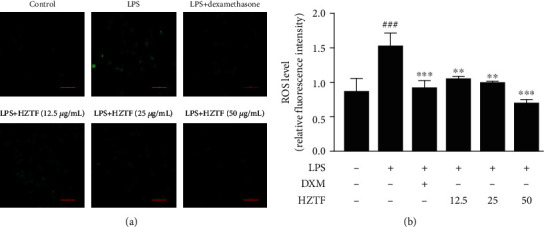
Effects of HZTF on LPS-induced ROS accumulation in RAW264.7 cells. After pretreatment with HZTF (12.5, 25, and 50 *μ*g/mL) or positive drug (dexamethasone at 10 *μ*g/mL) treatment for 2 h then treatment with LPS (100 ng/mL) for 24 h, cells were incubated with 10 *μ*M DCFH-DA for 30 min. (a) The fluorescent microscopy showing production of intracellular ROS (scale bar: 50 *μ*m). (b) The relative fluorescence intensity was analyzed by the Image J software (version 1.51). Data were presented as the mean ± SD (*n* = 3). ^###^*P* < 0.001, compared with the control group; ^∗∗^*P* < 0.01, ^∗∗∗^*P* < 0.001, compared with the LPS group.

To explore whether HZTF has an effect on oxidative damage, the level of ROS in RAW264.7 cells was measured using intracellular ROS probe, H2DCF-DA. As shown in Figure 5, we found that HZTF significantly (*P* < 0.01) suppressed the accumulation of intracellular ROS at all dosages tested (12.5, 25, and 50 *μ*g/mL), compared to the control group.

### 3.6. HZTF Suppressed mRNA Expression of Inflammatory Cytokine and Mediators

**Figure 6:**
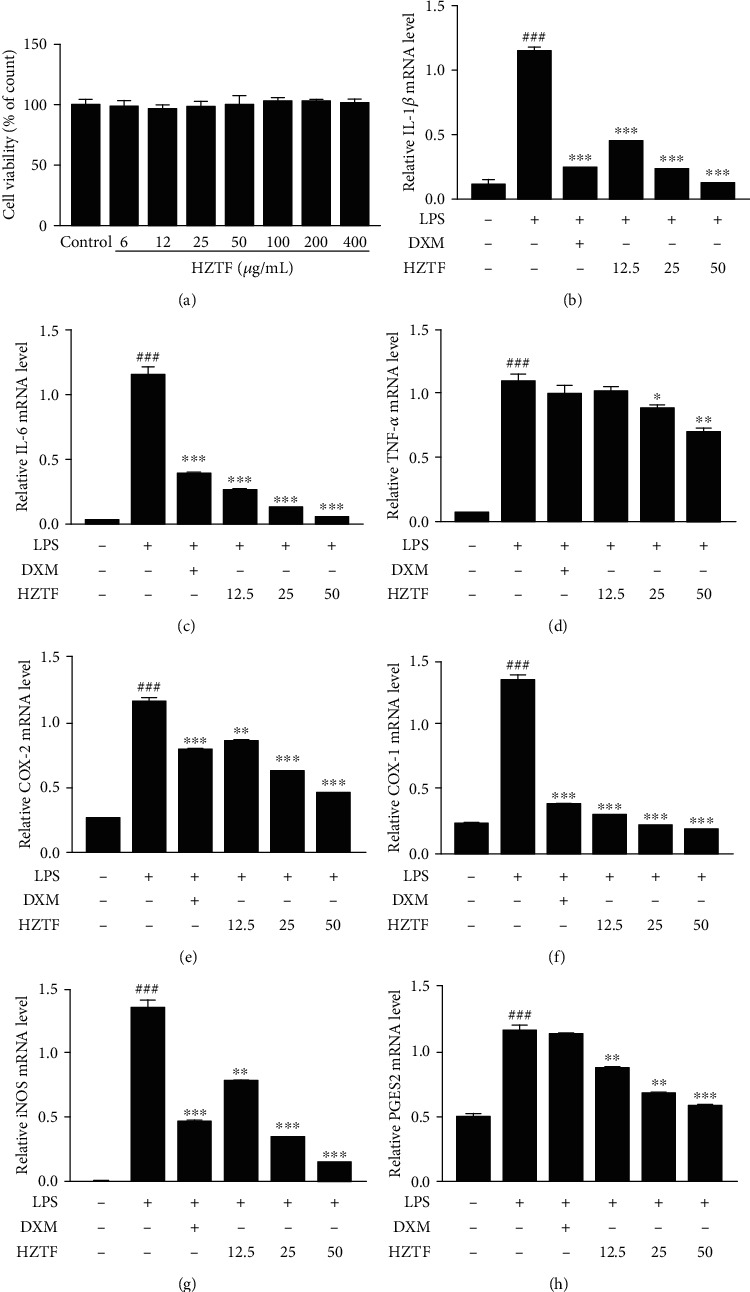
Anti-inflammatory effect of HZTF in LPS-stimulated RAW264.7 macrophages. (a) Cell viability as determined by the MTT assay. The relative mRNA expression of (b) IL-1*β*, (c) IL-6, (d) TNF-*α*, (e) COX-2, (f) COX-1, (g) iNOS, and (h) PGES2, following pretreated with different doses of HZTF for 2 h and stimulated by LPS (100 ng/mL) for 6 h. Results were presented as means ± SD (*n* = 3). ^###^*P* < 0.001 compared with the control group; ^∗^*P* < 0.05, ^∗∗^*P* < 0.01, and ^∗∗∗^*P* < 0.001 versus the LPS group.

To identify suitable doses and evaluate the effect of HZTF in the LPS-induced inflammation experimental model, the cytotoxicity of HZTF was examined. As shown in Figure 6(a), no obvious toxicity was observed in a serial concentration of HZTF from 6 *μ*g/mL to 400 *μ*g/mL. Hence, 12.5 (low dose), 25 (medium dose), and 50 *μ*g/mL (high dose) were determined as the treatment doses for our study.

It is widely acknowledged that mass inflammatory cytokine and mediators demonstrate a high expression level after inflammation induction, such as interleukin-1*β* (IL-1*β*), interleukin-6 (IL-6), tumor necrosis factor-*α* (TNF-*α*), cyclooxygenase-2 (COX-2), and inducible nitric oxide synthase (iNOS) [38–40]. Through pathway enrichment, the results revealed the strong regulation effect of HZTF on the arachidonic acid pathway. In the early mechanism study of HZTF on LPS-induced inflammation, the mRNA expression levels of IL-1*β*, IL-6, TNF-*α*, cyclooxygenase-1 (COX-1), COX-2, prostaglandin E synthase 2 (PGES2), and iNOS were measured. As shown in Figures 6(b)–6(f), LPS induced a significant increase in the mRNA expression of IL-1*β*, IL-6, TNF-*α*, COX-1, COX-2, PGES2, and iNOS, which indicated the occurrence of inflammation. Meanwhile, the LPS-induced inflammation was treated with different doses of HZTF. The aforementioned mRNA expression levels of the inflammatory cytokines and mediators were remarkably inhibited in a concentration-dependent manner. These results preliminarily indicate that HZTF could inhibit LPS-induced inflammation.

### 3.7. HZTF Decreased Production of Cytokine and Other Inflammatory Mediators

**Figure 7:**
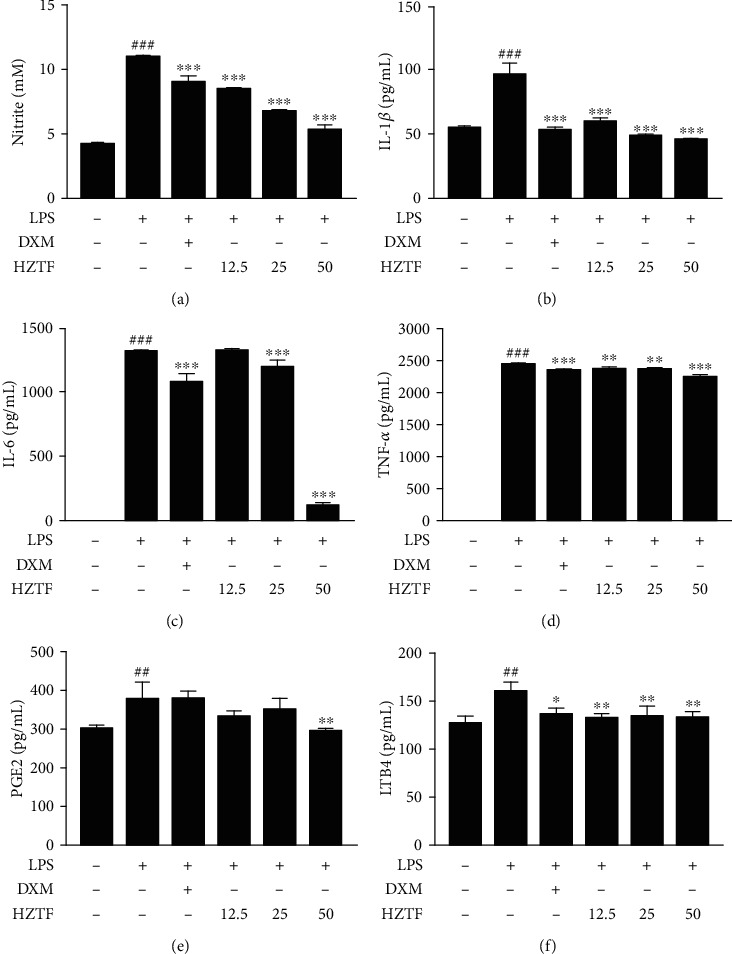
Effects of HZTF on the production of IL-1*β*, IL-6, TNF-*α*, PGE2, and LTB4 in RAW264.7 cells under stimulation of LPS. RAW264.7 cells were pretreated with HZTF for 1 h and then induced with LPS (100 ng/mL) for 24 h. The concentration of NO (a) was determined by the Griess assay. (b) IL-1*β*, (c) IL-6, (d) TNF-*α*, (e) PGE2, and (f) LTB4 level in the cell culture supernatant were quantified using an ELISA kit. The data were presented as means ± SD (*n* = 3). ^##^*P* < 0.01, ^###^*P* < 0.001 compared with the control group; ^∗^*P* < 0.05, ^∗∗^*P* < 0.01, and ^∗∗∗^*P* < 0.001 versus the LPS group.

To examine the effects of HZTF on the production of inflammatory cytokine and mediator in RAW264.7 cells stimulated by LPS, the concentrations of nitric oxide (NO), IL-1*β*, IL-6, TNF-*α*, prostaglandin E2 (PGE2), and leukotriene B4 (LTB4) in the culture supernatant of RAW264.7 macrophages were measured by ELISA. The results showed that levels of NO, IL-1*β*, IL-6, TNF-*α*, PGE2, and LTB4 in the LPS group were elevated (*P* < 0.01 or *P* < 0.001) comparing relative to the control group. However, these proinflammatory effects were reversed by HZTF treatment at the doses tested (Figure 7).

### 3.8. HZTF Modulated the Pathway of Arachidonic Acid

**Figure 8:**
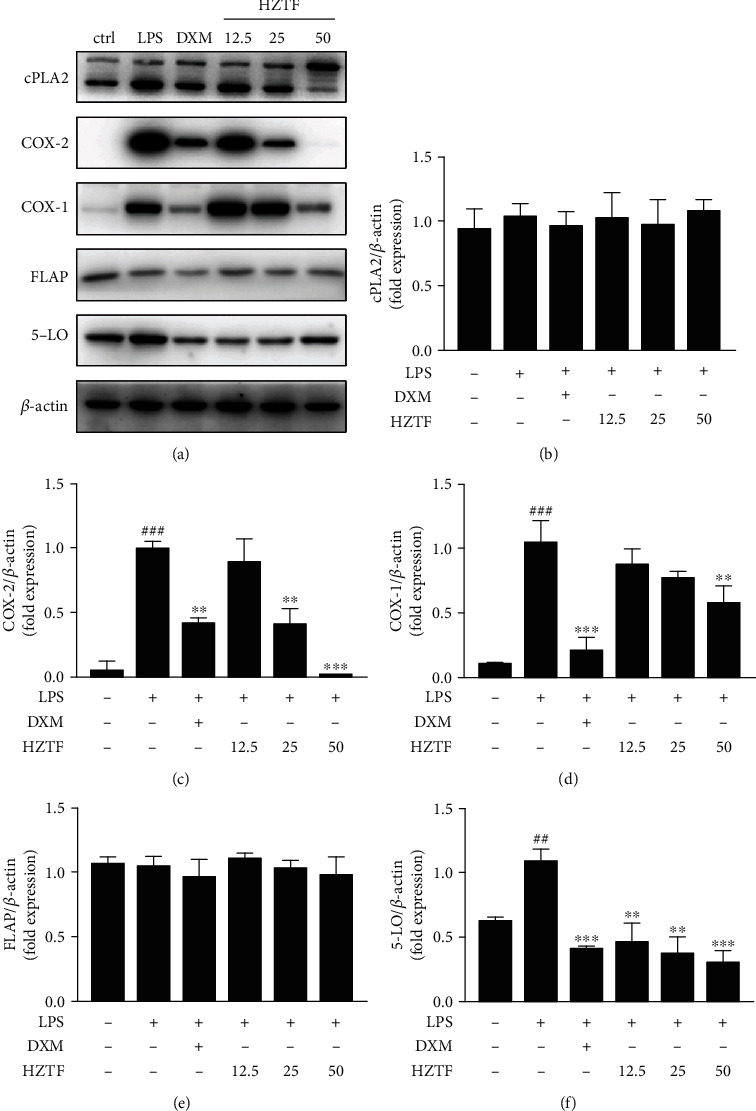
Alterations of the arachidonic acid (AA) pathway in the RAW264.7 cells following HZTF treatment: (a) expression of cPLA2, COX-2, COX-1, FLAP, and 5-LO by Western blot analysis; (b) the cPLA2/*β*-actin ratio; (c) the COX-2/*β*-actin ratio; (d) the COX-1/*β*-actin ratio; (e) FLAP/*β*-actin ratio; (f) the 5-LO/*β*-actin ratio. Sample loading was normalized by *β*-actin. *n* = 3; ^##^*P* < 0.01,^###^*P* < 0.001 vs. control group; ^∗∗^*P* < 0.01,^∗∗∗^P < 0.001 vs. LPS-induced group.

As shown in Figure 8, HZTF exhibited a significant inhibitory effect on COX 1 (Figures 8(a) and 8(d)), COX 2 (Figures 8(a) and 8(c)), and 5-LO (Figures 8(a) and 8(f)) in RAW264.7 cells stimulated by LPS, as demonstrated by Western analysis. However, it appeared that similar effect did not apply to the cPLA2 and FLAP expression (Figures 8(a), 8(b), and 8(e)).

## 4. Discussion

HZTF is an effective, but relatively new, TCM-based anti-GA therapy. In this study, in order to uncover the underlying mechanism of action of this formula HZTF in which multiple compounds are involved, we chosen to combine a network pharmacology approach with a focused experimental validation design (Figure 1). We successfully identified the core active ingredients of HZTF, constructed the compound target-disease target network, and predicted the potential metabolic pathway enrichment by mapping the compound targets with the disease targets. Importantly, we validated the predicted pathways involved in the pharmacological actions of HZTF, supporting that HZTF significantly suppressed the metabolism of arachidonic acid by inhibiting COX1, COX2, and 5-LO, leading to anti-inflammatory and analgesic effects for GA.

As stated previously, HZTF formula was developed based on the principles/theory used in TCM practice emphasizing treatment outcome. Despite established efficacy, very little information is available on the underlying mechanism of HZTF used as anti-GA therapy. Network pharmacology has recently been used as an effective tool to predict the underlying mechanism of TCM herbs or formula [23, 26]. Hence, in this study, we adopted a similar approach to examining the mechanism of action of HZTF. The DAVID database was used to predict pathways that are closely related to gouty disease, and the results showed that arachidonic acid metabolism, cytokine-cytokine receptor interaction, NOD-like receptor signaling pathway, and JAK-STAT signaling pathway are the most relevant predicted pathways. These results are in agreement with previously published data [41–44]. The most important finding of note in this study is the discovery that the arachidonic acid metabolism pathway is the number 1 shared signaling pathway of HZTF in GA as shown in Figure 4(d). Importantly, the inhibition of HZTF on arachidonic acid metabolism pathway was subsequently validated by a series of *in vitro* experiments.

Arachidonic acid is metabolized via catalysis of two enzymes COX and 5-LO. Metabolism through COX leads to formation of prostaglandins, causing pain, vasodilation (swelling and redness), and fever. When 5-LO is combined with its activating protein, FLAP, arachidonic acid undergoes a series of transformations and finally forms leukotriene, promoting neutrophil chemotaxis [45–47]. Under the gouty condition, sodium urate crystals are deposited on the joints, and inflammatory cells are recruited around them; ultimately inflammatory mediators such as PGE2, LTB4, and IL-1*β* are released causing symptom-related inflammatory reactions and pain responses [48]. PGE2 and LTB4 are the end products of the COX and 5-LO branches of arachidonic acid, respectively, and are closely related to the symptoms at gouty sites [49]. At the cellular level, we showed that HZTF significantly downregulated products derived from both COX (PGE2) and 5-LO (example being LTB4). We believe that the downregulation observed contributes to the efficacy of HZTF. Further, molecular biology experiments revealed that HZTF has significant inhibitory effects on the upstream proteins PGE2 and LTB4, as well as COX-1, COX-2, and 5-LO. The identified active ingredients from HZTF provide strong support for the HZTF's inhibitory effect on the arachidonic acid metabolism. It has been shown that ingredients such as quercetin, catechin, luteolin, rhein, sitosterol, baicalein, and stigmasterol have a suppressive effect on COX [50–53], while compounds including baicalein, quercetin, taxifolin, and caffeic acid were reported to downregulate 5-LO [54–57].

HZTF treatment leads to low-level recruitment of white blood cells in the synovium, significant antiswelling effect, and improvement of the pain threshold in the GA models. These phenomena are the direct result of the inhibition of the arachidonic acid metabolism pathway, leading to reduced formation of prostaglandins and leukotriene. The antioxidative effects of HZTF may partially account for the underlying mechanism. NO and ROS, which are affected by the activation of the arachidonic acid pathway [58, 59], were also significantly inhibited by HZTF as demonstrated in our *in vitro* experiments.

Although many previous studies have reported that the arachidonic acid metabolism pathway plays an important role in the pathogenesis of gout [60–62], very few herbal-based antigout therapies have been reported to have a mechanism of action associated with the inhibition of arachidonic acid pathway. Napagoda and colleagues showed that lipophilic extracts of *Leucas zeylanica* inhibited 5-LO, microsomal prostaglandin E2 synthase-1, and xanthine oxidase, rationalizing its application as anti-inflammatory and antigout remedy [15]. It appears that the reported underlying mechanisms for other TCM-based antigout therapies are mainly related to the inhibition of the NLRP3 pathway [13, 43, 63].

Currently, NSAIDs are the commonly used and recommended first-line therapy for GA patients [64]. It is well known that NSAIDs exert their pharmacological actions by blocking the rate limited enzyme COX to suppress the formation of prostaglandins. However, the common side effects of NSAIDs, such as gastrointestinal and renal toxicities, greatly limit its clinical use as the long term therapy for GA patients [65]. In contrast, no gastrointestinal adverse effect has been observed in any clinical trials using HZTF. Comparing the mechanisms of action for NSAIDs and HZTF, HZTF blocks all the enzyme limited metabolism pathways by blocking both COX and 5-LO, while NSAIDs only suppress COX. Previous studies demonstrated that by individually inhibiting COX-2, arachidonic acid metabolism would be shunted in the 5-LO pathway, producing increased amounts of 5-LO products such as leukotriene, leading to unwanted effects of NSAIDs. Coinhibition of COX and 5-LO would remove this shunt effect [66]. Our study provides direct evidence that HZTF simultaneously downregulates COX and 5-LO, which blocks the shunt effect in arachidonic acid metabolism. We speculate that this unique effect of HZTF may explain the reduced side effect profile of HZTF observed in the treatment of GA.

In addition to the inhibition of the arachidonic pathway, we found that HZTF has a broad anti-inflammatory property inhibiting a number of other inflammatory cytokine and mediators including IL-1*β*, IL-6, TNF-*α*, and iNOS, at both transcriptional and protein levels (Figures 6 and 7). Although we did observe a significant inhibition of TNF-*α* mRNA and protein in the experiments, the magnitude of TNF-*α* protein decrease in the cell culture supernatant (Figure 7(d)) was far less than that of mRNA (Figure 6(d)). This may be related to the timing of the experiment whereby the changes in TNF-*α* protein did not tightly correlated with the mRNA level within the experimental time frame. As shown in other studies, the level of TNF-*α* mRNA transcription did not always correlated to the release of TNF-*α* in RAW264.7 cell [67].

**Figure 9:**
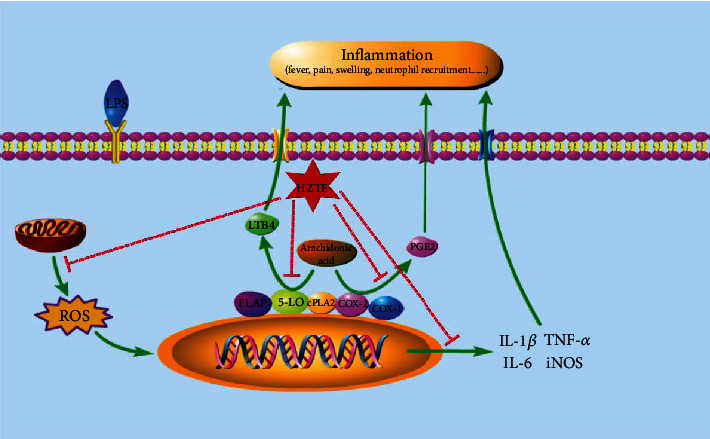
Overview of mechanism of HZTF on GA.

In general, the mechanism of action for any TCM recipe is complex and difficult to elucidate, due to multiple ingredients involved. In this study, we have successfully demonstrated that the key mechanism of HZTF as an anti-GA therapy is the inhibitory effect of HZTF on the metabolism of arachidonic acid pathway and on inflammatory mediators at both translational and protein levels, thus rationalizing the use of HZTF as an anti-GA therapy. This was completed through pathway prediction using network pharmacology and then *in vitro* and *in vivo* experimental verification. The possible mechanism and signaling pathway of HZTF are summarized in Figure 9.

There are some limitations in this study. Firstly, many active components of HZTF identified have anti-inflammatory effects and other related pharmacological actions; however, the importance of specific ingredients in this therapy remains unknown. Secondly, this study only explores the impact of HZTF on the key targets of the arachidonic acid pathway and gene and protein expression of some inflammatory mediators. Other downstream targets have not been investigated. Therefore, in the future, more in-depth studies exploring the detailed mechanism of action for HZTF are required to advance the understanding of the pharmacology of HZTF and to optimize the clinical application of HZTF therapy.

## 5. Conclusion

In conclusion, we have successfully used the network pharmacological analysis method combining “wet-laboratory” experiments to explore the pharmacological mechanisms of HZTF, a patented TCM formula for GA. Our study predicts the active ingredients in the 4 herbs of HZTF and the connection between multiple targets of the ingredients in herbal formula and multiple targets of GA. Based on the results of pathway enrichment, we verified that HZTF relieves the inflammatory response in GA by suppressing the arachidonic acid pathway and production of inflammatory cytokines and mediators such as NO, IL-1*β*, IL-6, TNF-*α*, PGE2, and LTB4. The superior clinical profile of HZTF featuring minimal adverse effects may be related to the characteristics of HTZF that simultaneously suppresses COX1, COX2, and 5-LO, delineating the differences in the mechanisms of action of NSAIDs and other herbal formulas.

## Figures and Tables

**Table 1:** Primers used in Real-Time PCR.

Gene	Forward primer	Reverse primer
IL-1*β*	GAGCCTGTGTTTCCTCCTTG	CAAGTGCAAGGCTATGACCA
IL-6	CTGACAATATGAATGTTGGG	TCCAAGAAACCATCTGGCTAGG
TNF-*α*	GGGAGCAAAGGTTCAGTGAT	CCTGGCCTCTCTACCTTGTT
COX-2	ATTCCAAACCAGCAGACTCATA	CTTGAGTTTGAAGTGGTAACCG
iNOS	GTTCTCAGCCCAACAATACAAGA	GTGGACGGGTCGATGTCAC
COX-1	ATGAGTCGAAGGAGTCTCTCG	GCACGGATAGTAACAACAGGGA
PGES2	CCTCGACTTCCACTCCCTG	TGAGGGCACTAATGATGACAGAG
*β*-actin	GGCTGTATTCCCCTCCATCG	CCAGTTGGTAACAATGCCATGT

**Table 2:** Active components identified from herbs of HZTF.

Herbs	Number	Components
Polygoni Cuspidati Rhizoma et Radix	10	6,8-Dihydroxy-7-methoxyxanthone, physovenine, picralinal, physciondiglucoside, torachrysone-8-O-beta-D-(6′-oxayl)-glucoside, rhein, sitosterol, (+)-catechin, luteolin, quercetin
Herba Plantaginis	7	Dinatin, baicalein, baicalin, sitosterol, 6-OH-luteolin, stigmasterol, luteolin
Ligustri Lucidi Fructus	8	Sitosterol, kaempferol, taxifolin, lucidumoside D, lucidumoside D_qt, eriodictyol, luteolin, quercetin
Nidus Vespae	9	Sitosterol, daucosterol, stearic acid, cerotic acid, protocatehuic acid, 4-hydroxybenzoic acid, caffeic acid, thymidine, p-dihydroxybenzene

**Table 3:** Effects of HZTF on white blood cells in rabbit knee joint effusion induced by MSU (*n* = 8).

Groups	Doses (g/kg/d)	WBC (×10^4^/mm^3^)
Model	—	11.40 ± 3.68
HZTF-L	0.20	9.46 ± 1.33^∗^
HZTF-M	0.35	8.16 ± 2.54^∗∗^
HZTF-H	0.70	7.16 ± 1.11^∗∗∗^

^∗^ *P* < 0.05, ^∗∗^*P* < 0.01, ^∗∗∗^*P* < 0.001, compared with the model group. HZTF-L: low dose of HZTF; HZTF-M: medium dose of HZTF; HZTF-H: high dose of HZTF.

**Table 4:** Effects of HZTF on xylene-induced ear swelling in mice (*n* = 10).

Groups	Doses (g/kg/d)	Ear swelling (mg)	Inhibition (%)
Model	—	10.2 ± 3.8	—
HZTF-L	0.25	8.4 ± 2.6^∗^	17.7
HZTF-M	0.50	5.8 ± 1.9^∗∗^	43.1
HZTF-H	1.00	4.4 ± 1.6^∗∗∗^	56.9

^∗^ *P* < 0.05, ^∗∗^*P* < 0.01, ^∗∗∗^*P* < 0.001, compared with the model group. HZTF-L: low dose of HZTF; HZTF-M: medium dose of HZTF; HZTF-H: high dose of HZTF.

**Table 5:** Effects of HZsTF on the time to respond in the hot-plate test in mice (*n* = 10).

Groups	Doses (g/kg/d)	Time to response (s)		
		30 min	60 min	90 min
Normal	—	16.6 ± 1.9	17.0 ± 2.3	17.8 ± 2.1
HZTF-L	0.25	22.5 ± 3.6^∗∗^	27.1 ± 3.0^∗∗^	23.0 ± 3.3^∗∗^
HZTF-M	0.50	22.9 ± 4.4^∗∗^	29.6 ± 4.4^∗∗^	24.7 ± 4.2^∗∗^
HZTF-H	1.00	27.4 ± 3.1^∗∗^	36.8 ± 4.3^∗∗∗^	27.2 ± 3.2^∗∗^

^∗∗^ *P* < 0.01, ^∗∗∗^*P* < 0.001, compared with the normal group. HZTF-L: low dose of HZTF; HZTF-M: medium dose of HZTF; HZTF-H: high dose of HZTF.

**Table 6:** Effects of HZTF on writhing response induced by antimony potassium tartrate in mice (*n* = 10).

Groups	Doses (g/kg/d)	Number of writhing	Inhibition (%)
Model	—	51.1 ± 11.4	—
HZTF-L	0.25	39.5 ± 6.4^∗∗^	22.7
HZTF-M	0.50	31.3 ± 5.2^∗∗∗^	38.7
HZTF-H	1.00	23.2 ± 6.4^∗∗∗^	54.6

^∗∗^ *P* < 0.01, ^∗∗∗^*P* < 0.001, compared with the model group. HZTF-L: low dose of HZTF; HZTF-M: medium dose of HZTF; HZTF-H: high dose of HZTF.

## Data Availability

The data used to support the findings of this study are available from the corresponding author upon request.

## Supplementary Materials

Supplementary Materials Table S1: detailed information of targets for HZTF compounds. Table S2: list of gout-related targets from disease databases. Table S3: list of 77 core targets extracted from a protein-protein interaction network. Table S4: list of KEGG pathway enrichment.
