# Supplementary material for: Chinese Medicine Huzhen Tongfeng Formula Effectively Attenuates Gouty Arthritis by Inhibiting Arachidonic Acid Metabolism and Inflammatory Mediators
Source: Mediators Inflamm. 2020 Oct 9;2020:6950206. doi: 10.1155/2020/6950206 (PMC7568794; doi:10.1155/2020/6950206)
Supplement: Supplementary Materials — Table S1: detailed information of targets for HZTF compounds. Table S2: list of gout-related targets from disease databases. Table S3: list of 77 core targets extracted from a protein-protein interaction network. Table S4: list of KEGG pathway enrichment. [file 6950206.f1.docx]

**Supplementary Materials**

**Table S1 | Detailed information of targets for HZTF compounds.**

| **Herbs** | **Active Compounds** | **Target Name** |
| --- | --- | --- |
| Polygoni Cuspidati Rhizoma et Radix | (+)-Catechin | PTGS1, PTGS2, HSP90AB1, HSP90AA1, PRKACA, RXRA, ESR1, NCOA2, CALM3, CALM2, CALM1, HAS2, CAT |
|  | 6, 8-Dihydroxy-7-Methoxyxanthone | ADRB2, PTGS1, PTGS2, HSP90AB1, HSP90AA1, PRKACA, DPP4, PIK3CG, CA2, GSK3B, CDK2, MAPK14, CHEK1, PKIA |
|  | Physciondiglucoside | TOP2A, TOP2B |
|  | Torachrysone-8-O-Beta-D-(6'-oxayl)-Glucoside | TOP2A, TOP2B |
|  | Physovenine | ADRB2, PTGS1, PTGS2, HSP90AB1, HSP90AA1, PRKACA, CA2, GSK3B, CDK2, NOS2, F2, NOS3, ACHE, PDE3A, RXRA, SLC6A2, SLC6A3, CHRM3, CHRM1, SCN5A, ADRA1A, CHRM2, ADRA1B, GABRA1, CHRNA7, AR, PRSS1, DRD1, CHRNA2, SLC6A4, OPRM1, ESR1, CCNA2, OPRD1, ADRA2B, ESR2, PIM1, GRIA2 |
|  | Picralinal | SCN5A, AR, OPRM1, OPRD1 |
|  | Rhein | PTGS1, PTGS2, HSP90AB1, HSP90AA1, PIK3CG, AKR1B1, NCOA2, JUN |
| Herba Plantaginis | 6-OH-Luteolin | PTGS1, PTGS2, HSP90AA1, HSP90AB1, NCOA2, PIK3CG, AR |
|  | Baicalein | PTGS1, PTGS2, HSP90AA1, HSP90AB1, NCOA2, PIK3CG, NCOA1, PRSS1, PRKACA, DPP4, CALM1, CALM3, CALM2, PDE3A, BCL2, FOS, BAX, HIF1A, FOSL1, FOSL2, CDK1, MPO, AHR, IGF2, CYCS, ALOX12, NFATC1, TDRD7, EGLN1, NOX5, FABP5, APOD, AR, RELA, AKT1, VEGFA, MMP9, CASP3, TP53, CCNB1 |
|  | Baicalin | F10, PTPN1 |
|  | Dinatin | PTGS1, PTGS2, HSP90AA1, HSP90AB1, NCOA2, PIK3CG, NOS2, F2, NOS3, ACHE, RHO, IGHG1, NCOA1, PRSS1, PRKACA, DPP4, CALM1, CALM3, CALM2 |
|  | Stigmasterol | PTGS1, PTGS2, NCOA2, IGHG1, NCOA1, PRKACA, PGR, CHRM3, CHRM1, SCN5A, HTR2A, ADRA1A, GABRA3, CHRM2, ADRA1B, ADRB2, GABRA1, CHRNA7, AKR1B1, NR3C2, ADH1C, RXRA, ADRA2A, SLC6A2, SLC6A3, PLAU, LTA4H, MAOB, MAOA, CTRB1, ADRB1 |
| Ligustri Lucidi Fructus | Eriodictyol | PTGS1, PTGS2, HSP90AA1, HSP90AB1, NCOA2, PIK3CG, PRKACA, HMOX1, NFE2L2, NQO1 |
|  | Kaempferol | PTGS1, PTGS2, HSP90AA1, HSP90AB1, NCOA2, PIK3CG, JUN, BCL2, BAX, PGR, CHRM1, CHRM2, ADRA1B, GABRA1, PRKACA, CASP3, GABRA2, NOS2, F2, NOS3, ACHE, CDK1, AHR, SLC6A2, AR, PRSS1, DPP4, RELA, AKT1, TNF, XDH, MMP1, HMOX1, ICAM1, GSTP1, SLC2A4, INSR, PPARG, TOP2A, TOP2B, F7, CALM3, CALM2, CALM1, IKBKB, AHSA1, MAPK8, NR1I2, CYP1B1, ALOX5, HAS2, PSMD3, NR1I3, DIO1, PPP3CA, GSTM1, GSTM2, AKR1C3, SLPI, STAT1, CYP3A4, CYP1A2, CYP1A1, SELE, VCAM1 |
|  | Lucidumoside D | F2 |
|  | Lucidumoside D_qt | PTGS2, HSP90AA1, HSP90AB1, NCOA2, ADRB2, SCN5A, NOS2, F2, ACHE, AR, PRSS1, DPP4, F10, ESR1, CA2, GSK3B, CDK2, CCNA2 |
|  | Taxifolin | PTGS1, PTGS2, HSP90AA1, HSP90AB1, PIK3CG, RELA, ICAM1, AKR1B1, RXRA, DGAT2, MTTP, APOB |
| Nidus Vespae | 4-Hydroxybenzoic Acid | TDP1, MAPT, CA12, CA1, CA2, CA3, CA4, CA6, CA5A, CA7, CA9, CA14, CA5B, CA13, APP |
|  | Caffeic Acid | TDP1, PTPN2, PTPN1, CA12, CA1, CA2, CA3, CA4, CA6, CA5A, CA7, CA9, CA14, CA5B, CA13 |
|  | Cerotic Acid | TDP1, FABP3, FABP4, FABP5, FABP12, FABP7, PMP2, FABP9, FABP2, CDC25A, CDC25B, MAPT, MBNL1, MBNL2, MBNL3 |
|  | Daucosterol | STAT3, STAT1, STAT2, STAT4, VDR, BCL2L1, BCL2, BCL2L2, AR, PTAFR, DRD2, CNR1, PTPN2, PTPN1, DRD4 |
|  | [p-Dihydroxybenzene](https://www.ncbi.nlm.nih.gov/pcsubstance/?term=%22p-Dihydroxybenzene%22%5bCompleteSynonym%5d%20AND%20785%5bStandardizedCID%5d) | TDP1, MAPT, CA12, CA1, CA2, CA3, CA5A, CA7, CA14, CA5B, CA13, APP, APLP2, IDO1, IDO2 |
|  | Protocatehuic Acid | TDP1, MAPT, CA12, CA1, CA2, CA3, CA4, CA6, CA5A, CA7, CA9, CA14, CA5B, CA13, COMT |
|  | Stearic Acid | TDP1, FABP3, FABP4, FABP5, FABP12, FABP7, PMP2, FABP9, FABP2, CDC25A, CDC25B, HSD11B1, HSD11B1L, PTGER2, MAPT |
|  | Thymidine | MAPT, TK1, TYMS, TYMP, FPGS, CDA, ADA, DCK, DGUOK, ADORA1, RNASE1, ANG, RNASE4, DTYMK, PYGL |
| Polygoni Cuspidati Rhizoma et Radix;  Herba Plantaginis;  Ligustri Lucidi Fructus | Luteolin | PTGS1, PTGS2, HSP90AB1, HSP90AA1, PRKACA, DPP4, PIK3CG, AR, PRSS1, TOP2A, NCOA2, JUN, CASP9, CASP3, RELA, EGFR, AKT1, VEGFA, CCND1, BCL2L1, CDKN1A, MMP2, MMP9, MAPK1, IL10, RB1, CDK4, TNF, IL6, TP53, NFKBIA, XDH, MDM2, APP, MMP1, PCNA, ERBB2, PPARG, HMOX1, CASP7, ICAM1, MCL1, BIRC5, IL2, CCNB1, TYR, IFNG, IL4, GSTP1, XIAP, SLC2A4, INSR, PTGES, NUF2, ADCY2, MET |
| Polygoni Cuspidati Rhizoma et Radix;  Ligustri Lucidi Fructus | Quercetin | ADRB2, PTGS1, PTGS2, HSP90AB1, HSP90AA1, PRKACA, DPP4, PIK3CG, F2, NOS3, ACHE, RXRA, SCN5A, GABRA1, AR, PRSS1, TOP2B, TOP2A, AKR1B1, NCOA2, JUN, BCL2, BAX, CASP9, CASP3, KCNH2, CASP8, PON1, TGFB1, HAS2, RELA, EGFR, AKT1, VEGFA, CCND1, BCL2L1, CDKN1A, MMP2, MMP9, MAPK1, IL10, RB1, TNF, IL6, TP53, NFKBIA, XDH, MMP1, ERBB2, PPARG, HMOX1, ICAM1, BIRC5, IL2, CCNB1, IFNG, GSTP1, SLC2A4, INSR, FOS, HIF1A, CDK1, MPO, AHR, IGF2, F10, PLAU, MAOB, F7, AHSA1, NR1I2, CYP1B1, ALOX5, PSMD3, NR1I3, DIO1, GSTM1, GSTM2, NFE2L2, NQO1, MMP3, EIF6, EGF, ELK1, POR, ODC1, RAF1, SOD1, STAT1, RUNX1T1, HSPA5, ACACA, CYP3A4, CYP1A2, CAV1, MYC, F3, GJA1, CYP1A1, IL1B, CCL2, SELE, VCAM1, PTGER3, CXCL8, PRKCB, DUOX2, HSPB1, SULT1E1, MGAM, PLAT, THBD, SERPINE1, COL1A1, PTEN, IL1A, NCF1, ABCG2, PARP1, COL3A1, CXCL11, CXCL2, DCAF5, CHEK2, CLDN4, PPARA, PPARD, HSF1, CRP, CXCL10, CHUK, SPP1, RUNX2, RASSF1, E2F1, E2F2, ACPP, CTSD, IGFBP3, IRF1, ERBB3, PCOLCE, NPEPPS, HK2, NKX3-1, RASA1 |
| Polygoni Cuspidati Rhizoma et Radix;  Herba Plantaginis;  Ligustri Lucidi Fructus;  Nidus Vespae | Sitosterol | ADRB2, PTGS1, PTGS2, HSP90AB1, HSP90AA1, PRKACA, PIK3CG, PDE3A, CHRM3, CHRM1, SCN5A, ADRA1A, CHRM2, ADRA1B, GABRA1, CHRNA7, DRD1, CHRNA2, SLC6A4, OPRM1, NCOA2, JUN, BCL2, BAX, PGR, HTR2A, GABRA3, CASP9, CASP3, KCNH2, GABRA2, CHRM4, GABRA5, CASP8, PON1, TGFB1, MAP2 |

**Table S2 | List of gout-related targets from disease databases.**

| **No.** | **Target name** |  | **No.** | **Target name** |  | **No.** | **Target name** |
| --- | --- | --- | --- | --- | --- | --- | --- |
| 1 | AKR1B1 |  | 76 | TTR |  | 151 | VDR |
| 2 | CXCR2 |  | 77 | CYP1A2 |  | 152 | PDZK1 |
| 3 | IL1B |  | 78 | UGT2B7 |  | 153 | ADIPOQ |
| 4 | ABCC1 |  | 79 | CYP2C18 |  | 154 | LEP |
| 5 | SLC22A6 |  | 80 | ALOX5 |  | 155 | TLR4 |
| 6 | PPARA |  | 81 | SCN4A |  | 156 | COMT |
| 7 | PPARG |  | 82 | ASIC1 |  | 157 | CXCL8 |
| 8 | PTGIR |  | 83 | KCNQ2 |  | 158 | NLRP3 |
| 9 | PTGS1 |  | 84 | KCNQ3 |  | 159 | TERT |
| 10 | PTGS2 |  | 85 | PLA2G2A |  | 160 | IL18 |
| 11 | XDH |  | 86 | SLCO1B1 |  | 161 | CCL2 |
| 12 | CXCR1 |  | 87 | SHBG |  | 162 | IL17A |
| 13 | PNP |  | 88 | CYP11B1 |  | 163 | P2RX7 |
| 14 | SLC22A12 |  | 89 | CYP11B2 |  | 164 | SLC22A11 |
| 15 | FABP1 |  | 90 | SERPINA6 |  | 165 | PON1 |
| 16 | SLC22A8 |  | 91 | HSD11B2 |  | 166 | SF1 |
| 17 | SLC22A7 |  | 92 | HSD3B1 |  | 167 | APOA1 |
| 18 | ABCG2 |  | 93 | SLCO2B1 |  | 168 | RELA |
| 19 | AOX1 |  | 94 | UGT1A3 |  | 169 | ALDH2 |
| 20 | UGT1A1 |  | 95 | UGT1A9 |  | 170 | IL4 |
| 21 | TAS2R16 |  | 96 | UGT2B4 |  | 171 | RREB1 |
| 22 | SLCO1C1 |  | 97 | BCL2 |  | 172 | KDR |
| 23 | SLCO1A2 |  | 98 | THBD |  | 173 | CST3 |
| 24 | SLC22A5 |  | 99 | CFTR |  | 174 | INHBC |
| 25 | SLC22A2 |  | 100 | GP1BA |  | 175 | S100A9 |
| 26 | SLC22A10 |  | 101 | S100A7 |  | 176 | EGF |
| 27 | SLC22A1 |  | 102 | AMACR |  | 177 | MAOA |
| 28 | SLC2A9 |  | 103 | CBR1 |  | 178 | PPARGC1A |
| 29 | SLC10A1 |  | 104 | GLO1 |  | 179 | RASGRP2 |
| 30 | ALB |  | 105 | PTGDR2 |  | 180 | ADH1B |
| 31 | PANX1 |  | 106 | CES1 |  | 181 | FCGR3A |
| 32 | ABCC6 |  | 107 | AKR1C3 |  | 182 | MEFV |
| 33 | ABCC5 |  | 108 | SERPINA7 |  | 183 | IL12B |
| 34 | ABCC4 |  | 109 | HSD11B1 |  | 184 | MYH9 |
| 35 | SLC16A7 |  | 110 | MAPK3 |  | 185 | APOC3 |
| 36 | SLC16A1 |  | 111 | PPARD |  | 186 | ADRB3 |
| 37 | CYP3A4 |  | 112 | AKR1B10 |  | 187 | NRXN2 |
| 38 | CYP2C8 |  | 113 | BCHE |  | 188 | IL23R |
| 39 | CYP2C19 |  | 114 | CD14 |  | 189 | S100A8 |
| 40 | ABCC3 |  | 115 | ALPK1 |  | 190 | MAP4K2 |
| 41 | ABCC2 |  | 116 | ALDH16A1 |  | 191 | CTSB |
| 42 | ABCC11 |  | 117 | CARD8 |  | 192 | TFAP2A |
| 43 | NR1I2 |  | 118 | HPRT1 |  | 193 | RELN |
| 44 | FABP2 |  | 119 | SLC17A3 |  | 194 | PKD2 |
| 45 | CYP2C9 |  | 120 | PRPS1 |  | 195 | LRP2 |
| 46 | CYP2B6 |  | 121 | ANKH |  | 196 | CDC42BPG |
| 47 | ABCB11 |  | 122 | UMOD |  | 197 | TRPM2 |
| 48 | NR3C1 |  | 123 | HNF1B |  | 198 | CXCL16 |
| 49 | ABCB1 |  | 124 | MUC1 |  | 199 | PYCARD |
| 50 | CYP19A1 |  | 125 | APRT |  | 200 | R3HDM2 |
| 51 | SCN10A |  | 126 | MAT1A |  | 201 | MAF |
| 52 | PTGER1 |  | 127 | G6PC |  | 202 | TBXAS1 |
| 53 | CYP2D6 |  | 128 | SEC61A1 |  | 203 | PPARGC1B |
| 54 | PDPK1 |  | 129 | HMCN1 |  | 204 | PRKG2 |
| 55 | CA2 |  | 130 | CFHR3 |  | 205 | SLC37A4 |
| 56 | CA3 |  | 131 | CFHR1 |  | 206 | TEC |
| 57 | ABCB5 |  | 132 | APOE |  | 207 | P2RY6 |
| 58 | MC2R |  | 133 | PFKM |  | 208 | PDK2 |
| 59 | CRH |  | 134 | SEC23B |  | 209 | WDR1 |
| 60 | HSD3B2 |  | 135 | DNAJB11 |  | 210 | KTN1 |
| 61 | CYP27B1 |  | 136 | REN |  | 211 | CYP4B1 |
| 62 | CYP24A1 |  | 137 | CBS |  | 212 | SLC28A2 |
| 63 | ANXA1 |  | 138 | XPNPEP3 |  | 213 | ABCF1 |
| 64 | NOS2 |  | 139 | CLCNKB |  | 214 | CLEC12A |
| 65 | NR0B1 |  | 140 | TBX19 |  | 215 | NRBP1 |
| 66 | CYP3A5 |  | 141 | LMNA |  | 216 | A1CF |
| 67 | CYP3A7 |  | 142 | LRP6 |  | 217 | PRPS2 |
| 68 | CYP11A1 |  | 143 | SLC17A1 |  | 218 | CARMIL1 |
| 69 | CYP17A1 |  | 144 | GCKR |  | 219 | BCAS3 |
| 70 | CYP1A1 |  | 145 | MEN1 |  | 220 | CLNK |
| 71 | CYP1B1 |  | 146 | TNF |  | 221 | SHLD2 |
| 72 | CYP2A6 |  | 147 | IL6 |  | 222 | ZNF518B |
| 73 | CYP2E1 |  | 148 | TGFB1 |  | 223 | IL17REL |
| 74 | CYP3A43 |  | 149 | IL10 |  | 224 | NIPAL1 |
| 75 | CYP4A11 |  | 150 | PYGM |  |  |  |

**Table S3 | List of 77 core targets extracted from protein–protein interaction network.**

| **No.** | **Target name** |  | **No.** | **Target name** |
| --- | --- | --- | --- | --- |
| 1 | APP |  | 40 | TLR4 |
| 2 | STAT3 |  | 41 | CYP2C8 |
| 3 | TNF |  | 42 | UGT2B7 |
| 4 | PTGS2 |  | 43 | UGT1A9 |
| 5 | CXCL8 |  | 44 | LEP |
| 6 | ALB |  | 45 | CYP2C19 |
| 7 | CYP3A4 |  | 46 | APOA1 |
| 8 | EGFR |  | 47 | ANXA1 |
| 9 | CYP1A1 |  | 48 | CASP3 |
| 10 | MMP9 |  | 49 | AR |
| 11 | IL1B |  | 50 | AKR1C3 |
| 12 | CYP2E1 |  | 51 | CYP2A6 |
| 13 | EGF |  | 52 | PPARG |
| 14 | IL10 |  | 53 | IL18 |
| 15 | STAT1 |  | 54 | DRD2 |
| 16 | RELA |  | 55 | CYP19A1 |
| 17 | MAPK3 |  | 56 | ADIPOQ |
| 18 | CYP1A2 |  | 57 | CNR1 |
| 19 | APOE |  | 58 | DRD4 |
| 20 | CYP3A5 |  | 59 | HSD3B1 |
| 21 | CYP2B6 |  | 60 | IL17A |
| 22 | CYP4A11 |  | 61 | TGFB1 |
| 23 | UGT1A1 |  | 62 | SLCO1B1 |
| 24 | ESR1 |  | 63 | CYP17A1 |
| 25 | CYP2C9 |  | 64 | HSD3B2 |
| 26 | IL4 |  | 65 | COMT |
| 27 | AHR |  | 66 | MAOA |
| 28 | ERBB2 |  | 67 | MMP3 |
| 29 | IDO1 |  | 68 | NR3C1 |
| 30 | CDK2 |  | 69 | PTGS1 |
| 31 | FABP4 |  | 70 | CYP1B1 |
| 32 | MAOB |  | 71 | OPRM1 |
| 33 | CDA |  | 72 | BCL2L1 |
| 34 | STAT4 |  | 73 | FABP1 |
| 35 | ABCG2 |  | 74 | CST3 |
| 36 | NR1I2 |  | 75 | HSD11B1 |
| 37 | ADORA3 |  | 76 | ADORA1 |
| 38 | CYP2J2 |  | 77 | PPARGC1A |
| 39 | MPO |  |  |  |

**Table S4 | List of KEGG pathway enrichment.**

| Term | Count | P Value | FDR |
| --- | --- | --- | --- |
| hsa00590:Arachidonic acid metabolism | 10 | 2.43×10^-8^ | 2.68×10^-5^ |
| hsa00150:Androgen and estrogen metabolism | 6 | 9.28×10^-5^ | 0.10 |
| hsa00380:Tryptophan metabolism | 6 | 1.36×10^-4^ | 0.15 |
| hsa04920:Adipocytokine signaling pathway | 6 | 1.53×10^-3^ | 1.67 |
| hsa03320:PPAR signaling pathway | 6 | 1.75×10^-3^ | 1.91 |
| hsa04060:Cytokine-cytokine receptor interaction | 10 | 5.64×10^-3^ | 6.04 |
| hsa04621:NOD-like receptor signaling pathway | 5 | 7.79×10^-3^ | 8.25 |
| hsa04620:Toll-like receptor signaling pathway | 6 | 9.01×10^-3^ | 9.49 |
| hsa04630:Jak-STAT signaling pathway | 7 | 13.7×10^-3^ | 14.1 |
| hsa04210:Apoptosis | 5 | 24.5×10^-3^ | 23.9 |
| hsa04660:T cell receptor signaling pathway | 5 | 48.5×10^-3^ | 42.2 |
